# Commercial polycarbonate track-etched membranes as substrates for low-cost optical sensors

**DOI:** 10.3762/bjnano.10.67

**Published:** 2019-03-07

**Authors:** Paula Martínez-Pérez, Jaime García-Rupérez

**Affiliations:** 1 Nanophotonics Technology Center, Universitat Politècnica de València, Camino de Vera s/n, 46022 Valencia, Spain

**Keywords:** chemical sensor, Fabry–Pérot interferometer, optical sensor, polycarbonate, track-etched membrane

## Abstract

Porous materials have become one of the best options for the development of optical sensors, since they maximize the interaction between the optical field and the target substances, which boosts the sensitivity. In this work, we propose the use of a readily available mesoporous material for the development of such sensors: commercial polycarbonate track-etched membranes. In order to demonstrate their utility for this purpose, we firstly characterized their optical response in the near-infrared range. This response is an interference fringe pattern, characteristic of a Fabry–Pérot interferometer, which is an optical device typically used for sensing purposes. Afterwards, several refractive index sensing experiments were performed by placing different concentrations of ethanol solution on the polycarbonate track-etched membranes. As a result, a sensitivity value of around 56 nm/RIU was obtained and the reusability of the substrate was demonstrated. These results pave the way for the development of optical porous sensors with such easily available mesoporous material.

## Introduction

Sensors are present in our daily life in order to detect and monitor chemical, biological and physical agents of interest in medical diagnosis, security, biodefense or industrial procedures, among other fields of application. For the design and fabrication of these sensing devices, huge efforts have been made in recent years to develop different transducers suitable for each application. According to the transducer type, sensors can be classified into several categories. Among all of them, optical sensors stand out because they exhibit a high sensitivity, the capability of multiplexing and direct real-time detection, miniaturization possibilities, immunity to electromagnetic interference and cost-effectiveness [1]. Additionally, by proper functionalization, label-free and specific detection can be achieved [2–4].

Optical sensors whose working principle is based on the detection of changes in the refractive index (RI) are the most widely used ones. Among them, those that base the sensing process on the interaction of an evanescent field with the target substance are the best known [2]. However, this kind of optical sensor presents a limited sensitivity, as only part of the light interacts with the substances of interest. To overcome this limitation, porous materials are a good option. Since they allow the recognition to happen inside the structure, the whole optical field interacts with the target substances. Furthermore, as the porous structure implies an increase in the surface-to-volume ratio, more receptors and, consequently, more analytes bind to the surface in biosensing applications. This allows the optical field to interact with much more matter, increasing the sensitivity as well [3,5].

Porous silicon is the most explored material for the fabrication of porous optical sensors. It can be easily fabricated and there are several well-known chemical strategies to modify its surface [6]. However, in recent years, new porous materials such as polymers [7] or metals [8] have attracted the attention of scientists. Nevertheless, all these porous materials require longer and more complex fabrication processes.

In this work, we propose an alternative, porous transducer that is commercially available for the development of optical sensors: polycarbonate track-etched (PCTE) membranes. PCTE membranes, which are typically used for size-based filtration [9–10], are reminiscent of the porous structure of a monolayer of porous silicon, a material that has an optical response of a Fabry–Pérot (FP) interferometer. This porous silicon structure has long been used for sensing [6] and we hypothesized that PCTE membranes might have the same optical response and be useful for sensing purposes, too.

To study the utility of PCTE membranes for sensing purposes, we characterized their optical response in the near infrared (NIR) region, optimized the material by placing a polished silicon surface under the PCTE membrane, and performed sensing experiments with different concentrations of ethanol. In this way, we have demonstrated for the first time to our knowledge that PCTE membranes are suitable for sensing RI variations. Therefore, this study provides a new porous transducer adequate for the development of optical sensors.

## Results and Discussion

### Optical response of PCTE membranes: a Fabry–Pérot interferometer

A FP interferometer is an optical structure consisting of two parallel reflective surfaces with a gap between them. When light travels through the structure, it reflects and generates interference that lead to the appearance of an interference fringe pattern.

This configuration has been emulated with porous silicon and has long been used for sensing purposes [6]. When porous silicon is illuminated an interference fringe pattern appears with maxima at particular wavelengths (λ*_m_*) given by the following formula:

[1] $$\lambda_{m}=\frac{2n_{\text{eff}}d}{m}\;,$$

where *m* is an integer, *d* is the layer thickness and *n*_eff_ is the effective RI of the porous layer [6]. When the porous structure is filled with a given substance or molecules, *n*_eff_ changes and λ*_m_* shifts, which is used to sense the presence of the substance or molecule.

**Figure 1:**
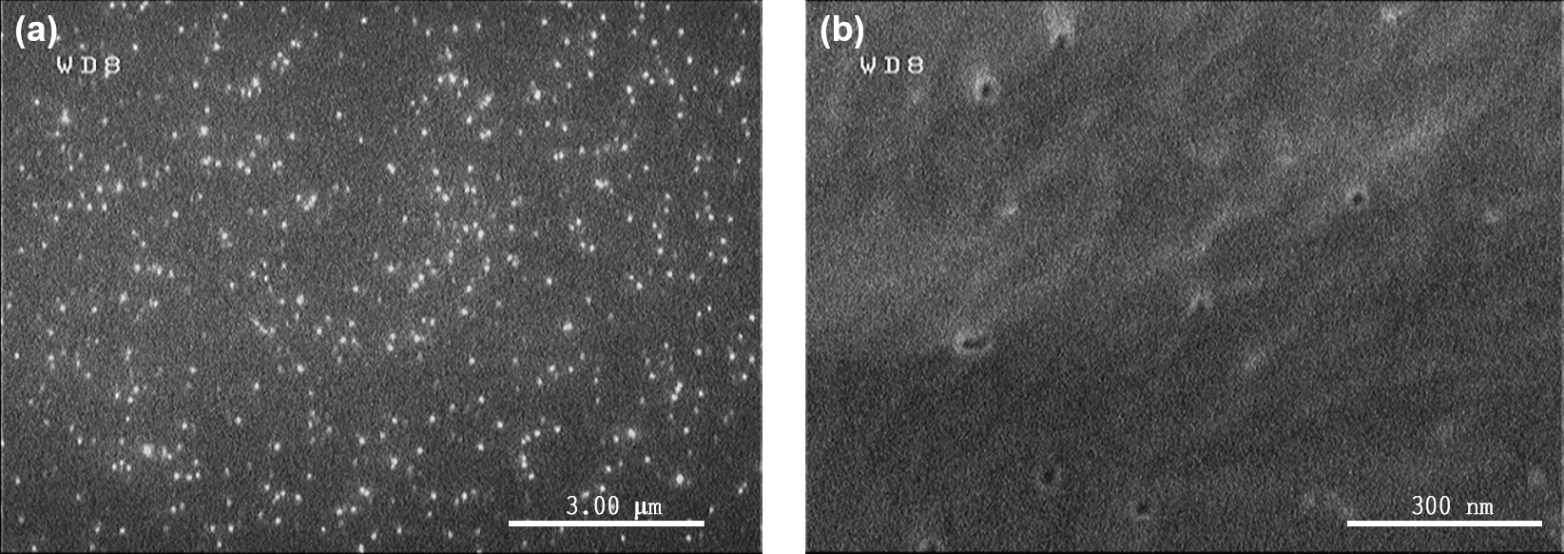
Field emission scanning electron microscope (FESEM) images of the surface of a PCTE membrane employed in our assays. (a) Randomly distributed pores cover the surface. (b) The diameter of the pores is around 30 nm, as indicated by the manufacturer.

In Figure 1, it can be seen that PCTE membranes have a porous structure similar to that exhibited by porous silicon. Therefore, we hypothesized that PCTE membranes could have an optical response similar to that of a FP interferometer and might be useful for sensing purposes as well.

**Figure 2:**
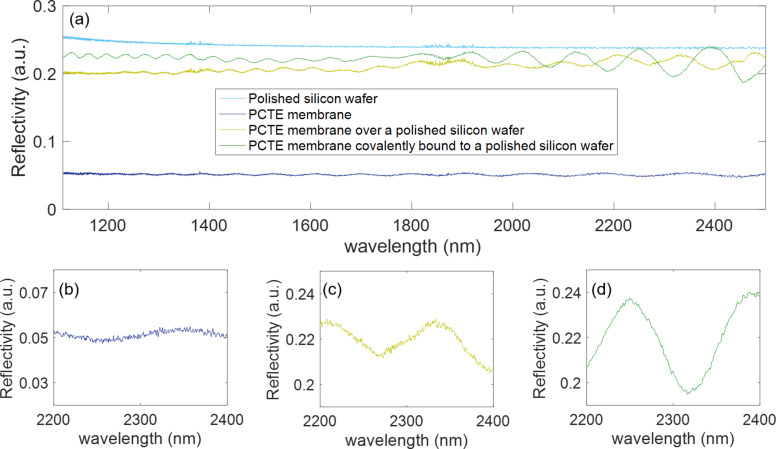
(a) Reflectivity spectra of PCTE membranes surrounded by air, positioned over a silicon substrate and covalently bound to it. Detail of the spectrum between 2200 and 2400 nm for the PCTE membrane (b) surrounded by air, (c) placed over silicon and (d) covalently attached to it. The same vertical scale has been used for the three spectra for a better comparison of the SNR.

Firstly, we checked if PCTE membranes exhibit an interference fringe pattern when exposed to light. For that aim, we performed vertical reflectivity measurements with an FTIR microscope (30 scans were taken for each measurement with a resolution of 4 cm^−1^) [3,7,11]. We effectively observed that an interference fringe pattern appears when the PCTE membrane is surrounded by air. However, the reflectivity was around 0.052 a.u., the peak-to-peak amplitude of the lobes was around 0.005 a.u. in the best case (see Figure 2a, dark blue curve), and the spectrum was notably noisy, with a signal-to-noise ratio (SNR) value of 3.52 (see Figure 2b). All these issues are disadvantageous for the future employment of these membranes as optical sensing structures for two reasons. First, the noise can hide tiny displacements of the spectra that could occur during the sensing event. Second, most solutions contain water and it is well known that its absorption coefficient in the NIR region is high [12]. If we place these aqueous solutions on the PCTE membrane in order to detect the presence of any component in it, part of the incident light will be absorbed by water and will not arrive to the PCTE membrane. This will cause the reflectivity signal to have an even lower intensity.

To maximize and improve the reflectivity signal, we placed the PCTE membranes on a polished silicon surface, which has a reflectivity signal of 0.24 a.u. and a flat spectrum in the NIR region where we are interested in carrying out the measurements (see Figure 2a, light blue curve). Furthermore, although other metals have a higher reflectivity, silicon offers an advantage: it might provide a mechanical support to the labile membrane. By means of the chemical protocol developed by Aran and co-workers [13], the PCTE membrane can be covalently attached to the silicon surface employing (3-aminopropyl)triethoxysilane (APTES) as a crosslinking reagent, which would avoid folding or displacement of the membrane during the sensing experiments.

When measuring the optical response of a PCTE membrane simply placed on silicon, i.e., not covalently bound, we observe that the reflectivity of the PCTE membrane increases up to 0.2 a.u. (a 4-fold increase), the peak-to-peak amplitude of the lobes increases up to 0.014 a.u. (see Figure 2a, light green curve) and the SNR improves up to 9.88 (see Figure 2c).

Finally, we studied the effect of the APTES-mediated covalent attachment of the PCTE membrane to the silicon substrate on the reflectivity spectrum. What we could conclude is that the APTES attachment preserves and even improves the reflectivity spectra of the PCTE membranes in order to perform sensing experiments. The reflectivity is around 0.2 a.u., the peak-to-peak amplitude of the lobes is around 0.044 a.u., which is better than the previous measurement with no APTES treatment, and the SNR increases up to 36.92 (see Figure 2d).

### Sensing of refractive index variations

Once it was demonstrated that the spectral response of the PCTE membranes is as expected (i.e., that for a FP interferometer) and that the covalent attachment to a silicon substrate improves the reflectivity spectrum and offers a mechanical support, our aim was to demonstrate their suitability for use in sensing applications. To this end, we placed a 10 µL drop of pure ethanol on the area of the PCTE membrane illuminated by the light beam of the FTIR microscope and let it evaporate at room temperature. We recorded the spectrum of the sample before the deposition of the drop and during the evaporation process every minute (i.e., the time required by the FTIR to perform a measurement with the configuration previously described). In this way, we can follow the shift experienced by the spectrum at a given point of the PCTE membrane in real time.

When the air present in the porous structure is replaced by ethanol, the *n*_eff_ of the structure increases, as ethanol has a higher RI than air. From Equation 1, we see that this is expected to provoke a shift of the spectrum towards longer wavelengths. Conversely, as ethanol evaporates, the air will start to fill the pores again and *n*_eff_ of the structure will become smaller, which should make the spectrum return to its initial position.

Regarding the spectrum, the best region to monitor how the position of its maxima changes when exposed to ethanol is between 2200 nm and 2500 nm. In this region, the peaks have a better quality factor and their amplitude is maximum, which will ultimately improve the sensitivity. In order to facilitate the identification of the peaks, the spectra were smoothed using the smooth function in MATLAB after acquisition.

**Figure 3:**
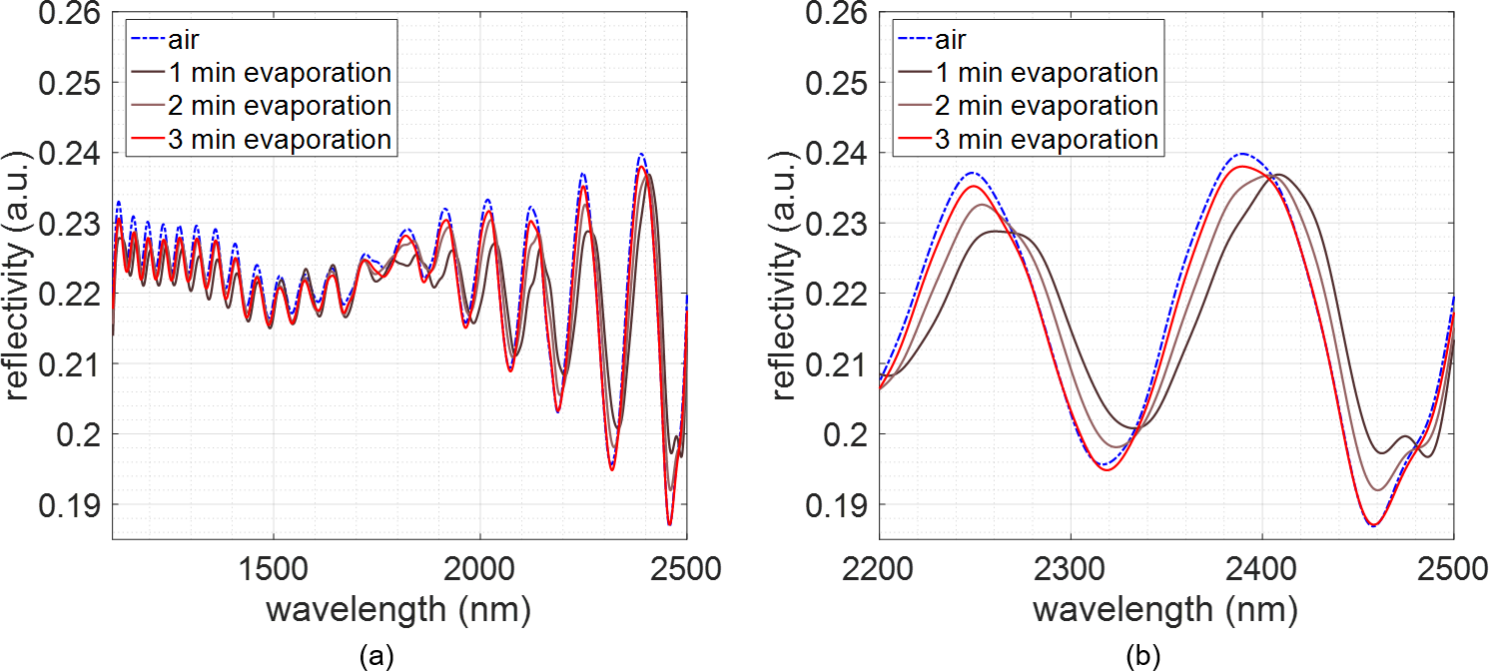
Evolution of the reflectivity spectrum of the PCTE membrane when placing pure ethanol on it. (a) For the whole characterization range (between 1110 and 2500 nm). (b) Detail of the spectral region between 2200 and 2500 nm.

In Figure 3b, we can see that when ethanol fills the pores a shift of the spectrum of 19.29 nm occurs towards longer wavelengths (for the maximum at ≈2400 nm). While evaporating, we can clearly see how the spectrum returns to its initial position gradually, and after 3 minutes, the sample seems to be almost dry as it reaches again the position in the beginning of the measurement. The differences in reflectivity intensity during the measurement process are due to the layer of liquid created on the top of the sample. It increases the diffuse component of light reflected by the sample, thus reducing the number of beams arriving to the lenses of the FTIR microscope.

**Figure 4:**
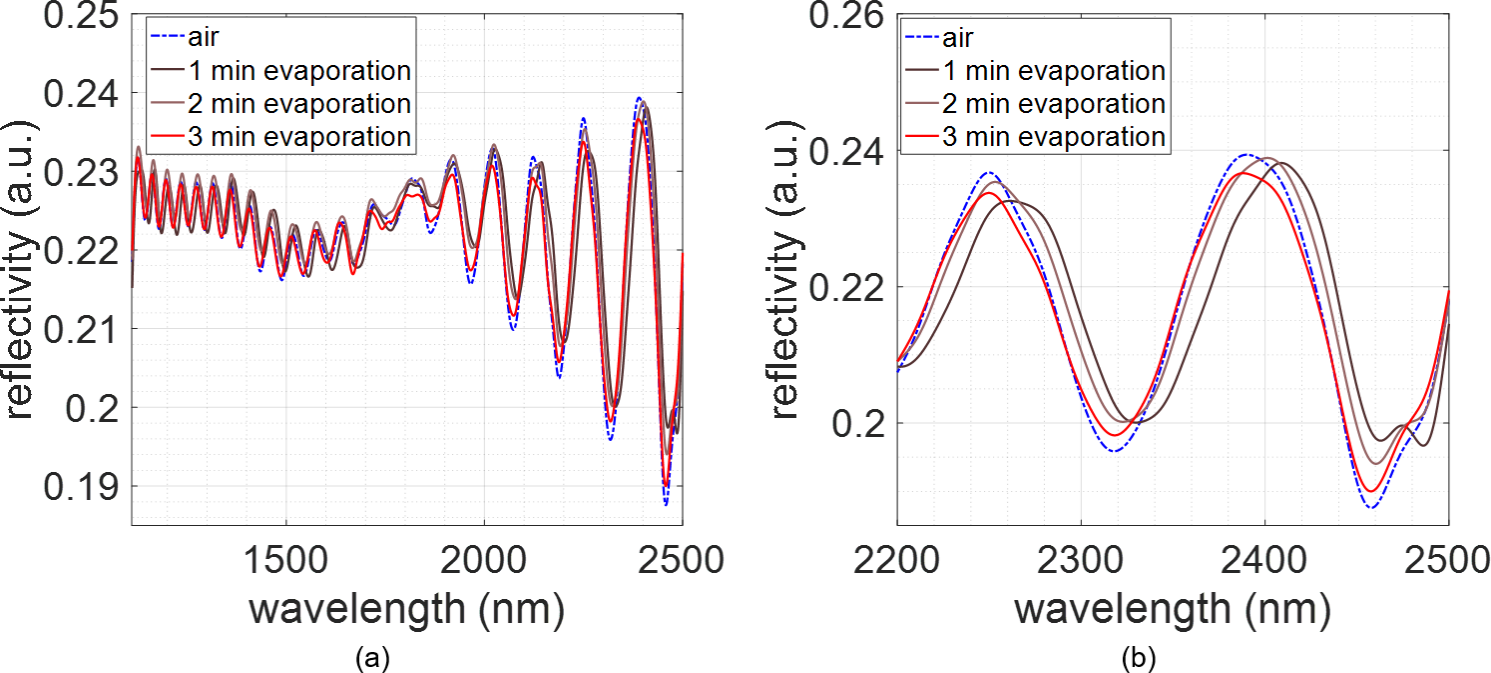
Evolution of the reflectivity spectrum of the PCTE membrane when placing a 50% ethanol solution on it. (a) For the whole characterization range (between 1110 and 2500 nm). (b) Detail of the spectral region between 2200 and 2500 nm.

Immediately after the sensing of pure ethanol, another 10 µL drop of 50% (v/v) solution of ethanol in water was placed on the PCTE membrane. We then repeated the previous process: we let the drop evaporate at room temperature and registered the spectrum every minute with the FTIR microscope. Figure 4 shows the reflectivity spectra measured while carrying out this experiment. Here we observe a shift of 18.34 nm for the maximum peak located at ≈2400 nm. Compared with the shift observed for pure ethanol, this shift is 0.95 nm smaller. The lower RI of the 50% ethanol solution, obtained by using the model proposed in [14], compared with the RI of pure ethanol obtained from [15], determines this smaller shift.

Nevertheless, the differences between the magnitudes of the two shifts with two different concentrations of ethanol might be larger. We also have to take into account that the spectrum was recorded every minute. Hence, we cannot assure that the shift registered for the first FTIR measurement after the deposition of the drop is the maximum shift achieved by the spectrum, as the liquid could have slightly evaporated during this time lapse [16] allowing the air to refill the pores. To check this, we calculated the sensitivity of our sensor (summarized in Table 1). As we performed both measurements on the same point of the sample and under the same conditions, the differences might only be attributed to the different evaporation behavior of the two solutions. For pure ethanol, we obtained a sensitivity of 56.49 nm/RIU, while for 50% ethanol solution we obtained 56.21 nm/RIU. Since they are very similar values, we can assume that the pores are equally filled by both solutions when the first FTIR measurement is done and that the differences in the shift only come from the different refractive index of the two solutions.

**Table 1:** Summary of the refractive index values for each substance filling the pores, measured spectral positions for them during the experiment and calculated sensitivity values.

Solutions	Refractive index (λ = 2400 nm)	Maximum position (nm)				Sensitivity (nm/RIU)
		Minute 0 (air)	Minute 1	Minute 2	Minute 3	

100% ethanol	1.3415	2389.49	2408.78	2403.42	2389.61	56.49
50% ethanol	1.3263	2390.09	2408.43	2401.28	2390.07	56.21
air	1.000					

These two sensing assays demonstrate the utility of PCTE membranes as optical sensors. The material behaves like a FP interferometer, whose spectral shift towards longer or shorter wavelengths depends on the increase or decrease of the RI of the medium, respectively. We could also see differences in the magnitude of those shifts depending on the RI of the liquid put on the sample. This indicates the capability of PCTE membranes not only to detect a change in the RI, but to quantify it.

Regarding sensitivity, our sensor is notably less sensitive than its homologous counterpart, porous silicon [5]. This might be explained by the fact that our sensors only have a 0.4% of porosity, while porous silicon typically has porosity in the range of 50%. However, even with such a low porosity value, we were able to clearly see the presence of ethanol in the medium. Furthermore, our porous structure presents an important advantage: it is ready to use. This material could reduce the costs and time of manufacturing. For future work, we will continue searching for a suitable way to improve the sensitivity of the device.

In this initial development stage, we tried to detect different concentrations of ethanol. Once we know we are able to use PCTE membranes for sensing chemicals, a wider study with different substances at different concentrations will be carried out. Furthermore, as polycarbonate surfaces can be chemically modified to bind molecules [17–19], this also paves the way to selectively detect analytes and to develop specific optical sensors for specific applications.

## Conclusion

For the first time, to our knowledge, we have demonstrated the utility of commercial PCTE membranes in the development of optical porous sensors. By means of the PCTE membranes, we could detect changes in the RI of the medium. Furthermore, we observed differences in the response depending on the magnitude of such changes, which indicates the utility of PCTE membranes not only for detection, but also for quantification.

This work means the discovery of a new, cheap and readily available transducer for the development of optical sensors. Moreover, as polycarbonate can be modified to be chemically reactive [17–19], this endows these optical sensors with versatility to fabricate devices for the selective detection and/or monitoring of chemical, physical or biological agents of different nature in different application fields.

## Experimental

All materials and reagents were purchased from Sigma-Aldrich (St. Louis, MO, USA), unless otherwise noted. Firstly, the surface morphology of Whatman^®^ 800307 PCTE membranes (19 mm diameter with pores of 30 nm diameter, 11 µm thickness and a refractive index of 1.5551 at 2400 nm (obtained using the dispersion equation provided in [20])) was characterized with a FESEM Hitachi S-4500 (Hitachi, Ltd., Chiyoda, Japan). In order to bind the PCTE membranes to a 1 × 1 cm polished silicon surface we slightly modified the APTES protocol previously described by Aran and co-workers [13]. Briefly, the PCTE membrane was firstly activated by oxygen plasma in a plasma asher (PVA TEPLA 200, PVA TePla AG, Wettenberg, Germany) for 1 min (50 W, 1.5 mbar). Immediately after, it was immersed in an aqueous solution of APTES at 80 °C for 20 min. Then, the membrane was removed from the solution and dried out on a cleanroom wipe. Once dried, it was dropped onto the newly activated silicon surface by piranha treatment (H_2_SO_4_/H_2_O 3:1) for 10 min.

A Bruker FTIR microscope (Bruker Corporation, Billerica, MA, USA) was employed to measure the optical response of the PCTE membranes in air and when different concentrations of ethanol (Scharlab, Barcelona, Spain) are placed on it. The reflectivity measurements were performed in the NIR range (1110–2500 nm) with a resolution of 4 cm^−1^. To enhance the SNR, 30 scans were collected every minute to perform a continuous monitoring of the spectrum shift evolution. For the fitting and the graphical representation of the spectra, MATLAB R2016b (The MathWorks, Inc., USA) was used.

## References

[1] Zhang Y-n, Zhao Y, Zhou T, Wu Q (2018). Lab Chip.

[2] Ruiz-Tórtola Á, Prats-Quílez F, González-Lucas D, Bañuls M-J, Maquieira Á, Wheeler G, Dalmay T, Griol A, Hurtado J, Bohlmann H (2018). J Biophotonics.

[3] Caroselli R, Martín Sánchez D, Ponce Alcántara S, Prats Quilez F, Torrijos Morán L, García-Rupérez J (2017). Sensors.

[4] Prabowo B A, Purwidyantri A, Liu K-C (2018). Biosensors.

[5] Caroselli R, Ponce-Alcántara S, Prats Quilez F, Martín Sánchez D, Morán Torrijos L, Griol Barres A, Bellieres L, Bandarenka H, Girel K, Bondarenko V (2017). Opt Express.

[6] Levitsky I A (2015). Sensors.

[7] Ponce-Alcántara S, Martín-Sánchez D, Pérez-Márquez A, Maudes J, Murillo N, García-Rupérez J (2018). Opt Mater Express.

[8] Qiu H-J, Li X, Xu H-T, Zhang H-J, Wang Y (2014). J Mater Chem C.

[9] Shindell O, Mica N, Ritzer M, Gordon V D (2015). Phys Chem Chem Phys.

[10] Párraga-Niño N, Quero S, Ventós-Alfonso A, Uria N, Castillo-Fernandez O, Ezenarro J J, Muñoz F-X, Garcia-Nuñez M, Sabrià M (2018). Talanta.

[11] Martín-Sánchez D, Ponce-Alcántara S, Martínez-Pérez P, García-Rupérez J (2019). J Electrochem Soc.

[12] Wilson R H, Nadeau K P, Jaworski F B, Tromberg B J, Durkin A J (2015). J Biomed Opt.

[13] Aran K, Sasso L A, Kamdar N, Zahn J D (2010). Lab Chip.

[14] García-Rupérez J, Toccafondo V, Bañuls M J, García Castelló J, Griol A, Peransi-Llopis S, Maquieira Á (2010). Opt Express.

[15] Sani E, Dell'Oro A (2016). Opt Mater.

[16] Ooi C H, Bormashenko E, Nguyen A V, Evans G M, Dao D V, Nguyen N-T (2016). Langmuir.

[17] Ogończyk D, Jankowski P, Garstecki P (2012). Lab Chip.

[18] Kosobrodova E, Jones R T, Kondyurin A, Chrzanowski W, Pigram P J, McKenzie D R, Bilek M M M (2015). Acta Biomater.

[19] Godeau G, Amigoni S, Darmanin T, Guittard F (2016). Appl Surf Sci.

[20] Sultanova N G, Kasarova S N, Nikolov I D (2013). Opt Quantum Electron.

